# A remote care model for patients at high risk of hospital admission due to COVID-19 deterioration: who makes it at home? – a multicenter follow-up case from Slovenia

**DOI:** 10.3325/cmj.2023.64.170

**Published:** 2023-06

**Authors:** Matic Mihevc, Diana Podgoršek, Jakob Gajšek, Samanta Mikuletič, Vesna Homar, Marko Kolšek, Marija Petek Šter

**Affiliations:** 1 Department of Family Medicine, Medical Faculty University of Ljubljana, Ljubljana, Slovenia; 2 Community Health Centre Ljubljana, Primary Healthcare Research and Development Institute, Ljubljana, Slovenia; 3 Primary Health Center Trebnje, Trebnje, Slovenia; 4 Primary Health Center Vrhnika, Vrhnika, Slovenia; 5 Primary Health Center Postojna, Postojna, Slovenia

## Abstract

**Aim:**

To assess the feasibility of a remote care model for high-risk COVID-19 patients, identify risk factors for hospital admission, and propose modifications to the tested model.

**Methods:**

We conducted a multicenter observational study of 225 patients (55.1% male) treated at three primary care centers between October 2020 and February 2022. Patients were enrolled into a telemonitoring program if they had a mild-moderate course of COVID-19 confirmed by polymerase chain reaction testing and were classified as high-risk for COVID-19 deterioration. Patients measured their vital signs three times daily, consulted their primary care physician every other day, and were followed up for 14 days. At inclusion, data were collected with a semi-structured questionnaire, and blood was drawn for laboratory analysis. A multivariable Cox regression model was used to determine predictors of hospital admission.

**Results:**

The median age was 62 years (range 24-94). The hospital admission rate was 24.4%, and the mean time from inclusion to hospital admission was 2.7 ± 2.9 days. A total of 90.9% of patients were hospitalized within the first five days. A Cox regression model, adjusted for age, sex, and the presence of hypertension, revealed that the main predictors of hospital admission were type-2 diabetes (hazard ratio [HR] 2.38, 95% confidence interval [CI] 1.19-4.77, *P* = 0.015) and thrombocytopenia (HR 2.46, 95% CI 1.33-4.53, *P* = 0.004).

**Conclusion:**

Telemonitoring of vital signs is a feasible method of remote care that helps identify patients requiring immediate hospital admission. For further scale-up, we suggest shortening call intervals in the first five days, when the risk of hospital admission is highest, and giving special attention to patients with type-2 diabetes and thrombocytopenia at inclusion.

The global health care systems have been greatly affected by the COVID-19 pandemic, experiencing significant disruptions due to the rapid spread of the virus, its unpredictable nature, and the emergence of new virus variants (1).

COVID-19 severity varies among patients and is influenced by different virus variants (1-3). The initial variants, namely Alpha, Gamma, and Delta, resulted in mild to moderate cases in 80% of patients, while 15% experienced severe symptoms and 5% developed critical disease (1-3). Even individuals with initially mild forms of the disease had a 15%-20% chance of progressing to more severe illness (1-3). In the later variants, particularly Omicron, which dominated in 2022, there was a higher proportion of milder forms of disease (4). Advanced age and underlying chronic conditions such as cardiovascular disease, type-2 diabetes, and chronic respiratory disease further increase the risk of severe COVID-19 (5-9).

During the pandemic, primary health care faced several challenges, including disrupted continuity of care, communication difficulties, and limited face-to-face interactions (5,6). To overcome these obstacles and ensure effective patient monitoring and support while ensuring the safety of health care workers, remote care models incorporating telemonitoring have been implemented (1,5,6).

Telemonitoring plays a crucial role by enabling remote monitoring of patients' symptoms and vital signs. It facilitates early detection of deteriorating conditions such as silent hypoxemia, where low blood oxygen levels may be present without noticeable symptoms or significant shortness of breath (6-9). Through diligent patient monitoring, health care providers can promptly intervene and prevent further deterioration. Furthermore, telemonitoring helps optimize the allocation of health care resources, ensuring timely care for those most in need (6-9).

However, successful integration of telemonitoring into clinical workflows depends on various factors, including the specific target population, disease trajectory, adherence to local and international guidelines, and compliance with regulatory frameworks (10-12). Several remote care models tailored for COVID-19 patients have been introduced (Table 1), encompassing synchronous (10,13-15) or asynchronous (16,17) telemonitoring of vital signs, combined with teleconsultations, or symptom-based monitoring alone (11,15,18). Risk assessment tools have been utilized to identify patients at higher risk of deterioration, leading to adjustments in consultation frequency as necessary (11,13). Typically, the monitoring period lasted for 10-14 days (10,11).

**Table 1:** Overview of initial COVID-19 remote care models*

Author	Healthcare level	Type of intervention	Selection criteria	Frequency of consultations
Silven et al (15)	Tertiary	Vital signs TM + video TC	Mild-moderately ill in need of close follow-up	Once daily
Blazey Martin et al (16)	Primary	Symptoms (±vital signs) phone TC	All patients stratified into high- or low-risk group	Once or twice daily
Crane et al (18)	Primary/tertiary	Vital signs TM + nurse phone TC	All patients stratified into high- or low-risk group	Twice daily
Shah et al (19)	Tertiary	Pulse oximetry TM + phone TC	All patients	Once daily
Annis et al (20)	Primary/tertiary	Symptoms monitoring + TC if needed	All patients	Once daily
Delgado et al (23)	Primary	SMS based symptoms monitoring + nurse phone TC if needed	All patients	Twice daily

*Abbreviations: TM – telemonitoring; TC – teleconsultation.

Throughout the COVID-19 pandemic, primary health care centers in Slovenia fulfilled their role as gatekeepers, providing care for individuals with mild to moderate manifestations of the disease. However, a distinct national protocol for managing high-risk patients susceptible to deterioration and subsequent hospital admission was lacking. As a result, inspired by successful models from previous studies, we developed a simple and scalable remote care model that specifically incorporates vital sign monitoring for high-risk COVID-19 patients. The aim of this study was to assess the feasibility of the remote care model for high-risk COVID-19 patients, identify risk factors for hospital admission, and propose modifications to the tested model.

## Participants and methods

We conducted a prospective, multicenter, observational cohort study in three primary care centers in Slovenia (Trebnje, Vrhnika, Postojna), involving mainly suburban and rural populations. The study was approved by the Medical Ethics Committee of the Republic of Slovenia and complied with the Declaration of Helsinki.

The study population included 225 patients who met the inclusion criteria and received a telemedicine package in the three primary care centers between October 2020 and February 2022.

Inclusion criteria were (a) an infection with SARS-CoV-2 confirmed through polymerase chain reaction (PCR) testing, (b) a mild to moderate course of COVID-19, (c) a high-risk for deterioration as assessed by the patient’s primary care physician, and (d) the ability to independently measure vital signs or having a caregiver available to assist with the measurements.

The high-risk criteria were (a) age ≥60 years, (b) the presence of chronic comorbidities such as cardiovascular disease, pulmonary disease, diabetes, oncologic disease, or other chronic conditions as determined by the primary care physician, and (c) exhibiting more severe symptoms of COVID-19 that could potentially lead to deterioration (chest pain, dyspnea, diarrhea with vomiting, and high fever). Patients displaying clinical signs of pneumonia without hypoxemia were also classified as high-risk. These criteria were established based on initial reports identifying predictors of worse outcomes in COVID-19 patients (19-22).

### Intervention

Enrolled patients were given a COVID-19 telemedicine package, which included a pulse oximeter (Rossmax SB220®, Rossmax, Taipei, Taiwan), a measurement protocol, and a measurement diary. They were instructed to measure their oxygen saturation level (SpO_2_), heart rate, and body temperature three times a day and document the readings in the measurement diary. The primary care physician provided guidance on the proper use of the pulse oximeter before its utilization.

Physicians established critical thresholds for vital signs, and patients were instructed to immediately contact the emergency number if their measurements exceeded these limits. The limits were individually determined based on the patient's comorbidities.

Patients received regular telephone check-ups from their primary care physician every other day to report their vital signs and symptoms until either their symptoms resolved, or hospital admission was required. If the recorded measurements fell within the reference ranges, the physician documented them in the medical records and provided further instructions for ongoing care. However, if the patient's condition deteriorated, they were promptly referred to a hospital.

### Integration of the intervention into established clinical pathways

**Figure 1:**
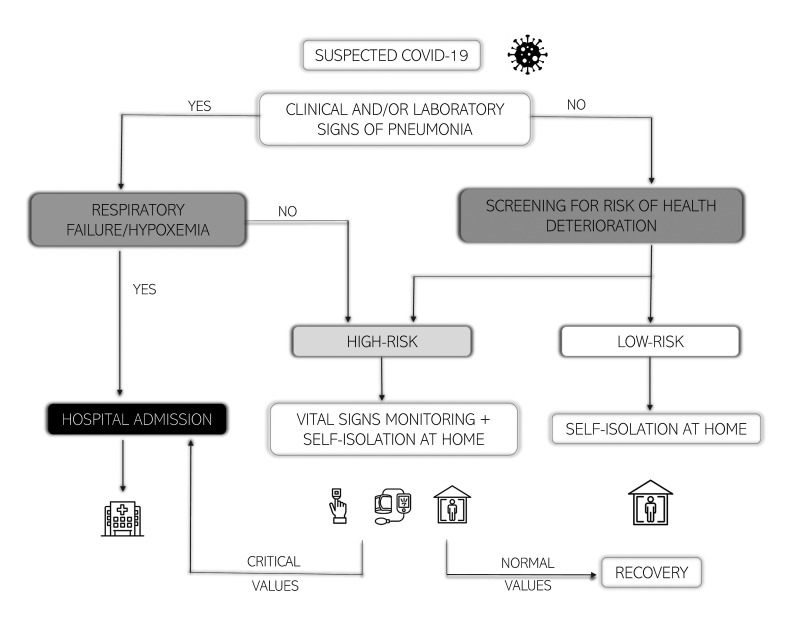
Management of patients with COVID-19 in the primary health care centers.

Primary-care centers assessed individuals with suspected COVID-19 and conducted a PCR test to confirm the presence of SARS-CoV-2 infection (Figure 1). Patients showing indications of pneumonia and respiratory failure were promptly referred to the hospital for further evaluation and care. Meanwhile, patients without clinical signs of pneumonia were categorized into two groups based on their risk of deterioration: low-risk and high-risk. High-risk patients were provided with a telemedicine package and were continuously monitored until their symptoms improved or until hospital admission was required. Low-risk patients were instructed to self-isolate at home.

### Observed variables and data sources

We collected variables from five categories (Table 2). At inclusion, patients completed an entry questionnaire, and a blood sample was obtained for laboratory analysis. After completion of telemonitoring, patients were called by phone and asked for follow-up information, which were later verified in the medical records and the health center information system.

**Table 2:** List of observed variables

Category	Data variable
Demographic data	age
	sex
Epidemiologic history	date of onset of symptoms
	date of positive polymerase chain reaction test
Clinical history	associated diseases
	body mass index
Laboratory values at inclusion	complete blood count
	C-reactive protein level
	creatinine level
	aspartate aminotransferase and alanine aminotransferase levels
Follow-up information	duration of telemonitoring
	time to hospital admission

### Statistical analysis

The normality of distribution was tested with a Shapiro-Wilks test. Numeric variables are reported as means and standard deviations, or medians with minimum and maximum values. Categorical variables are presented with absolute and relative frequencies. Baseline differences between groups were assessed with a two-tailed independent samples *t* test or a Mann-Whitney U test. A χ^2^ test was employed for categorical variables.

To identify the predictors of hospital admission within 14 days of inclusion, survival analysis and a multiple Cox proportional hazards model were used. The hazard function represents the risk of being hospitalized due to COVID-19 deterioration at a given time (23). Patients were followed up from the date of inclusion until hospital admission or were censored 14 days after inclusion if no hospital admission occurred.

Initially, Kaplan-Meier curves and the log-rank test were employed to compare the probability of hospital admission between groups. Subsequently, a univariate Cox proportional hazards model was used to estimate the relative increase in hazard for hospital admission based on the presence or absence of the variables of interest. Variables with a *P* value of less than 0.05 were considered potentially suitable for inclusion in a multivariable model (23). However, the final selection of variables of interest was based on previous reports, which identified confounding factors, as well as professional knowledge (19-22,24).

Finally, four multivariable Cox proportional hazards models were created to examine associations between variables while controlling for the selected confounders, namely sex, age, and body mass index. Age and body mass index were treated as continuous variables to maximize the amount of information obtained. The level of statistical significance was set at a *P* value of less than 0.05. Statistical analysis was conducted with IBM SPSS Statistics for Windows, version 25.0 (IBM Corp., Armonk, NY, USA).

## RESULTS

### Baseline characteristics

The patients’ (N = 225) baseline clinical data are presented in Table 3. Upon inclusion, significant differences were observed between patients who were hospitalized and those who were not in terms of age, presence of chronic diseases (arterial hypertension, type-2 diabetes, dyslipidemia, chronic kidney disease, previous deep vein thrombosis/pulmonary embolism), thrombocyte count, C-reactive protein level, and aspartate aminotransferase level (Table 3, Table 4). The mean time from onset of symptoms to assessment in the primary care clinic (which corresponds to the duration of disease at baseline) was 5.3 ± 3.5 days.

**Table 3:** Comparison of clinical characteristics between hospitalized and non-hospitalized COVID-19 patients at inclusion*

Baseline characteristics	All patients (N = 225)	Non-hospitalized patients (N = 170)	Hospitalized patients (N = 55)	t-value/ χ2- value	*P*
Age, years, median (min, max)	62 (24, 94)	58 (24, 94)	68 (43, 92)	4.039	<0.001
Men (%)	55.1	54.1	58.2	0.277	0.598
Arterial hypertension (%)	53.3	48.2	69.1	7.262	0.007
Type-2 diabetes (%)	16.9	12.9	29.1	7.721	0.005
Dyslipidemia (%)	19.6	16.5	29.1	4.207	0.040
Cardio-vascular disease (%)	17.8	15.3	25.5	2.935	0.087
Chronic obstructive pulmonary disease (%)	6.2	5.3	9.1	1.027	0.311
Asthma (%)	5.3	4.7	7.3	0.542	0.461
Chronic kidney disease (%)	15.1	12.4	23.6	4.124	0.042
Previous deep vein thrombosis/pulmonary embolism (%)	3.1	1.8	7.3	4.182	0.041
Body mass index, kg/m^2^, mean ± standard deviation	29.8 ± 5.4	29.2 ± 5.1	31.0 ± 5.4	1.825	0.070

**Table 4:** Comparison of laboratory characteristics between hospitalized and non-hospitalized COVID-19 patients at inclusion

Laboratory variables	All patients (N = 225), median (min, max)	Non-hospitalized patients (N = 170), median (min, max)	Hospitalized patients (N = 55), median (min, max)	U-value	p
Hemoglobin level (g/L)	137 (68, 169)	137 (99, 166)	134 (68, 169)	1824.5	0.302
Thrombocyte count (10^9^/L)	194 (17, 658)	212 (93, 450)	170 (17, 658)	1364.5	0.003
C-reactive protein level (mg/L)	30 (1, 263)	18 (1, 210)	44 (1, 263)	1447.5	0.001
Creatinine level (μmol/L)	84 (51, 834)	78 (51, 360)	85 (52, 834)	1023.5	0.946
Aspartate aminotransferase level (μkat/L)	0.56 (0.24, 3.02)	0.46 (0.24, 1.69)	0.71 (0.27, 3.02)	287.0	0.004
Alanine aminotransferase level (μkat/L)	0.56 (0.20, 3.52)	0.45 (0.20, 2.38)	0.63 (0.24, 3.52)	429.5	0.341

### Follow-up data and predictors of hospitalization

The hospital admission rate was 24.4%. On average, patients were hospitalized within 2.7 ± 2.9 days from the inclusion, with the hospital admission period ranging from 0 to 11 days. The majority of hospital admissions (90.9%) occurred within the first five days, and the latest was observed on the 11th day from inclusion. Importantly, no patients were lost to follow-up during the study period.

**Figure 2:**
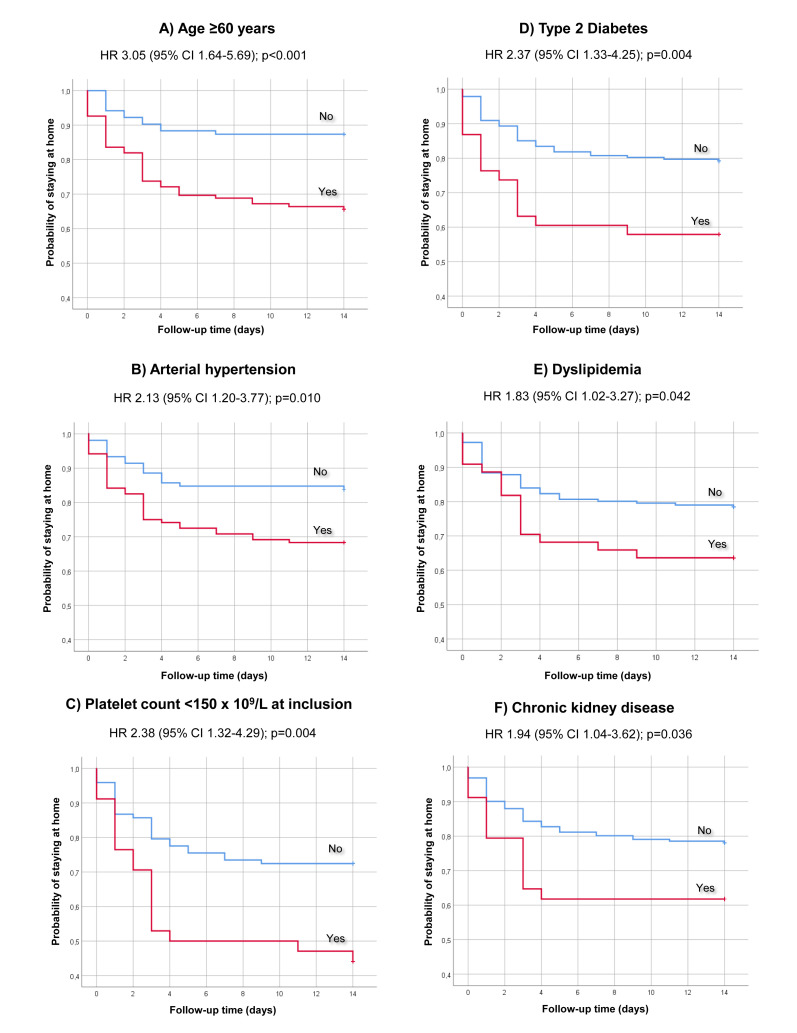
Kaplan-Meier curves of the probability of staying at home during the first 14 days depending on the presence of (**A**) age ≥60 years, (**B**) arterial hypertension, (**C**) platelet count <150 × 10^9^/L at inclusion, (**D**) type-2 diabetes, (**E**) dyslipidemia, and (**F**) chronic kidney disease. HR – hazard ratio; CI – confidence interval.

In the univariate Cox regression analysis, six factors were significantly associated with a higher hazard of hospital admission: age ≥60 years, arterial hypertension, platelet count <150 × 10^9^/L at inclusion, type-2 diabetes, dyslipidemia, and chronic kidney disease (Figure 2).

In the multivariable Cox regression analysis (Table 5), four models were created and controlled for three confounding factors (sex, age, body mass index). In all four models, type-2 diabetes was a significant predictor of hospital admission. In model 4, in which body mass index was excluded as a confounding factor, platelet count <150 × 10^9^/L at inclusion was also independently predictive of 14-day hospital admission.

**Table 5:** Multivariable Cox proportional hazard models with variables of interest and confounders (sex, age, body mass index)

	MODEL 1: all variables		MODEL 2: no sex		MODEL 3: no age		MODEL 4: no body mass index	
	HR (95% CI)	*P*	HR (95% CI)	*P*	HR (95% CI)	*P*	HR (95% CI)	*P*
Male sex	1.53 (0.73-3.20)	0.261	/	/	1.48 (0.71-3.10)	0.300	1.23 (0.65-2.35)	0.526
Age (continuous)	1.02 (0.99-1.06)	0.136	1.02 (0.99-1.05)	0.151	/	/	1.02 (0.99-1.05)	0.066
Body mass index (continuous)	1.02 (0.95-1.11)	0.576	1.03 (0.95-1.11)	0.541	1.01 (0.93-1.09)	0.837	/	/
Arterial hypertension	1.08 (0.46-2.52)	0.869	1.03 (0.44-2.38)	0.954	1.36 (0.61-3.04)	0.456	1.10 (0.56-2.16)	0.783
Type-2 diabetes	2.72 (1.20-6.15)	0.016	2.42 (1.11-5.29)	0.027	3.43 (1.59-7.39)	0.002	2.38 (1.19-4.77)	0.015
Platelet count <150 × 10^9^/L	2.08 (0.98-4.45)	0.058	1.95 (0.93-4.11)	0.079	2.11 (0.99-4.46)	0.051	2.46 (1.33-4.53)	0.004

*Abbreviations: HR – hazard ratio; CI – confidence interval.

## DISCUSSION

In contrast to previous research focusing on non-monitored individuals, this study specifically investigated the risk factors within the monitored population, thereby providing valuable insights directly applicable to the remote care model employed.

Previous studies have already established the efficacy and acceptability of telemonitoring systems in supporting COVID-19 patients in primary care and hospital settings (6,11,15,18,25). These systems have proven successful in detecting rapid health deterioration, including silent hypoxemia, and have shown promise in reducing emergency department visits and facilitating post-hospital discharge (25-28).

The study findings revealed that most patients sought help toward the end of the first week after experiencing COVID-19 symptoms, which coincided with the pulmonary phase of the disease (1,3). Consequently, any eligible patient had the potential for rapid deterioration (3,19,21). Approximately one in four patients was at risk of hospital admission, with particular attention required within the initial five days, as the majority of hospital admissions occurred within this timeframe. These findings align with previous research reporting hospital admissions occurring between four to eight days from inclusion (6,14). However, the proportion of hospital admissions varied across studies, ranging from 6% to as high as 20%, depending on the inclusion of high-risk criteria (11,18,27).

The study identified type-2 diabetes and thrombocytopenia as significant risk factors for hospital admission within a 14-day period. These findings align with previous research that has recognized type-2 diabetes as an independent predictor of hospital admission in COVID-19 patients (19,21,22,24). Chronic hyperglycemia contributes to worse outcomes by impairing immune function, increasing susceptibility to the virus, and promoting chronic inflammation (21,22,29). Thrombocytopenia, frequently observed in COVID-19 patients, has also been linked to poorer outcomes, especially in moderate or severe cases (30-32). The development of thrombocytopenia involves complex processes such as bone marrow suppression, platelet destruction, and involvement in the coagulation cascade (30). Although the study only assessed platelet counts at the time of inclusion, previous research has highlighted the significance of monitoring platelet counts as indicators of multi-organ failure and in-hospital mortality among COVID-19 patients (31,32). Furthermore, the study's Cox regression models revealed an interaction between body mass index and platelet count. High body mass index can influence platelet characteristics, which may explain why platelet count alone did not emerge as a significant predictor of hospital admission when considering both factors together (33).

Based on the study findings, recommendations were made to modify the remote care model. These recommendations include implementing daily follow-up calls for the first five days and gradually reducing the frequency to every other day from days 6 to 10, a step allowing close monitoring during the critical early phase and adjusting the monitoring frequency as the risk of hospital admission decreases. Delegating follow-up calls to nursing staff can help address the shortage of primary care physicians and ensure effective patient management. Additionally, expanding the high-risk criteria to incorporate laboratory parameters such as thrombocyte count would enhance the accurate identification of high-risk patients.

The telemonitoring model used in this study is scalable and suitable for primary care and clinical settings. It offers advantages such as low startup costs and compatibility with existing clinical pathways (12,16,17), which distinguish it from more complex and resource-intensive systems (10,13-15). However, challenges related to training requirements and increased workload for health care professionals conducting follow-up calls need to be addressed for successful implementation and sustainability (6,17,30).

The study limitations include its non-randomized design and lack of a control group. Predictors of hospital admission were only analyzed at baseline, and further laboratory testing could provide additional insights. The exclusion of patients unable to use a pulse oximeter may have affected the results, although family members were encouraged to assist with SpO_2_ measurements. Furthermore, the reliability of reported temperatures was influenced by patients using their own thermometers at home. However, since temperature was not a critical factor for hospital admission, we considered this limitation when developing our model. Finally, while the study focused on high-risk populations, telemonitoring programs have demonstrated positive cost-effectiveness outcomes even in low-risk populations, a finding suggesting potential benefits for a broader range of COVID-19 patients (28,34,35).

In conclusion, the greatest challenge for primary care physicians during the COVID-19 pandemic was to identify patients at high risk of COVID-19 deterioration and subsequent hospital admission. The implementation of a telemonitoring protocol in primary care centers proved feasible and facilitated the continuous care of patients while remotely identifying those in need of immediate hospitalization. As the pandemic subsides, there is a compelling need to extend the application of this model to manage other acute respiratory diseases and exacerbations of chronic diseases such as asthma, chronic obstructive pulmonary disease, or heart failure. By adapting and transferring the telemonitoring protocol to these diverse health care scenarios, we can enhance patient management and improve outcomes in a wide range of health care settings.
